# 

**Published:** 1916-09

**Authors:** 


### CONTENTS.

ORIGINAL COMMUNICATIONS—

Pyelitis in Infancy; A Reportt of a Case and Review of Current Literature—by John F. Jenkins, M. D., Opelika, Ala. ...... 245

Stone Impacted in the Lower End of the Ureter—by Courtney W. Shropshire, M. D. and Chas. Waterson, M. D., Birmingham, Ala. .................. 254

Army Medical Corps Examination ......... 261

EDITORIAL ......................... 262

SELECTIONS AND ABSTRACTS .......... 264

